# Zika Virus: Transmission, Detection, Control, and Prevention

**DOI:** 10.3389/fmicb.2017.00110

**Published:** 2017-02-03

**Authors:** Anshika Sharma, Sunil K. Lal

**Affiliations:** School of Science, Monash UniversitySelangor, Malaysia

**Keywords:** *Flavivirus* infection, arbovirus, sylvatic cycle, microcephaly, Guillain-Barré syndrome, Zika diagnosis

## Abstract

Zika virus (ZIKV) is a mosquito-borne *Flavivirus* discovered in Uganda in the 1940s. To date, three major ZIKV outbreaks have been reported. ZIKV infections have known to be primarily asymptomatic while causing mild illness in a few cases. However, the recent emergence and spread of ZIKV in the Americas has resulted in the declaration of “Public Health Emergency of International Concern” due to the potential association between the infection and prenatal microcephaly or other brain anomalies. In Brazil, a 20-fold increase in prenatal microcephaly cases and 19% increase in Guillain-Barré Syndrome (GBS) cases were reported in 2015, as compared to the preceding year. The probable deleterious effects of ZIKV infection prompt the urgent development of diagnostics and therapeutics. To this end, the existing evidences supporting the increasingly common prenatal microcephaly and GBS association and the current known ZIKV transmission dynamics, modes of detection (molecular and serology-based), and current control strategies are summarized in this review. This review also emphasizes the importance of understanding ZIKV transmission in order to design a sensitive yet cost and time-efficient detection technique. Development of an efficient detection technique would subsequently allow for better surveillance and control of ZIKV infection. Currently, limited literature is available on the pathogenesis of ZIKV, hence, focusing on the modes of ZIKV transmission could potentially contribute to the understanding of the disease spectrum and formulation of targeted treatment and control.

## Introduction to zika virus

Zika virus (ZIKV), a mosquito-borne *Flavivirus* belonging to the *Flaviviridae* family, is an emerging pathogen that is spreading rapidly across the Americas, raising concerns in the forefront of global healthcare (Ayres, 2016). The virus is closely related to other members of the *Flavivirus* genus (positive-sense, single-stranded RNA viruses), including the dengue virus (DENV), West Nile virus (WNV), yellow fever virus (YFV), tick-borne encephalitis virus (TBEV), and Japanese encephalitis virus (JEV) (Lazear and Diamond, 2016). Although, ZIKV infection is reported to be subclinical in approximately 80% of the cases, the virus has recently raised a “Public Health Emergency of International Concern” due to the dramatic increase in the cases of prenatal microcephaly and Guillain-Barré Syndrome (GBS) in ZIKV endemic regions (Basarab et al., 2016). Microcephaly is characterized by at least two standard deviation reduction in brain volume intellectual and motor disabilities, and behavioral issues (Petersen L. R. et al., 2016). Multiple development factors, such as genetic, environmental, and infectious exposure, during pregnancy are known to contribute to the onset of prenatal microcephaly. Therefore, further efforts are required toward eliminating any potentially associated confounding factor (Weaver et al., 2016). GBS, on the other hand, is a rare autoimmune disorder of the peripheral nervous system which could result in muscle weakness, paralysis, or even death (Lazear and Diamond, 2016). The plausible association between ZIKV infection and prenatal microcephaly/GBS is yet a matter of debate among researchers. It was suggested that the rise in the number of prenatal microcephaly and GBS cases could potentially be attributed by increased awareness and/or misdiagnosis (Mlakar et al., 2016).

To date, the exact ZIKV transmission dynamics have not been established. ZIKV has been isolated from humans, non-human primates, and multiple species of mosquitoes, suggesting a complex transmission network (Lazear and Diamond, 2016). Investigation of other potential inter-human modes of ZIKV transmission, such as sexual, blood-related, or maternal transmission, has allowed refinement of precautionary measures for ZIKV prevention and novel modes of ZIKV detection. Greater understanding of ZIKV transmission dynamics could also further aid in the development of a precise, rapid and simple tests for ZIKV detection in humans and mosquitoes. The robust detection method could in-turn improve the control of ZIKV, further preventing it from spreading around the globe (Pardee et al., 2016). With the severity of ZIKV associated diseases and the urgent need to develop methods to control its spread, in this review we aim to provide consolidated up-to-date available information on ZIKV associated prenatal microcephaly and GBS, ZIKV transmission dynamics, current molecular and serology-based modes of ZIKV detection, and the latest ZIKV control strategies in place.

### Epidemiology

ZIKV was first isolated from the blood of a febrile sentinel rhesus monkey in the Zika Forest of Uganda in 1947 (Dick et al., 1952; Lanciotti et al., 2008; Haddow et al., 2012). In the following years, ZIKV was isolated from various species of *Aedes* mosquitoes (Mlakar et al., 2016; Slavov et al., 2016). The first case of ZIKV infection in human was reported in Nigeria in 1954 (MacNamara, 1954). Since then, a number of significant outbreaks have been reported, prominently in the African and Southeast Asian region (Pan American Health Organization, 2016c).

The first major ZIKV outbreak occurred in 2007 on the Yap Island, Federated States of Micronesia, with approximately 75% of the population being affected within a period of 4 months (Saiz et al., 2016). Subsequently, in 2013 and 2014, ZIKV epidemic was reported in the French Polynesia, Cook Islands, Ester Islands, and New Caledonia. In 2015, ZIKV outbreak was reported in Brazil and henceforth has spread across the Latin America, Caribbean, and other parts of the world causing a pandemic (Lazear and Diamond, 2016). As of March 2016, ZIKV had spread to 33 countries in the Americas, with approximately over 1.5 million cases reported (Ayres, 2016; Petersen L. R. et al., 2016). By July 7th 2016, autochthonous cases of ZIKV had been reported in 40 countries within the Americas (Pan American Health Organization, 2016b). The exact global prevalence of ZIKV infection has not been reported due to the absence of a standardized protocol for differential diagnosis and clinical resemblance to other *Flavivirus* infections. In addition, ZIKV is known to be self-limiting (asymptomatic in approximately 80% of the cases), hence, it is likely that the infection is underdiagnosed/underreported in a disease-endemic setting (Gourinat et al., 2015).

It is anticipated that ZIKV would further spread around the globe, particularly via viremic travelers or the movement of infected mosquitoes (Petersen L. R. et al., 2016). More recently, autochthonous ZIKV transmission as well as cases of ZIKV transmission via sexual activity were reported in the United States (Centers for Disease Control and Prevention, 2016b; Hills et al., 2016). Currently, autochthonous vectorial ZIKV transmission has not been reported in Europe, although imported and locally sexually transmitted cases have been reported (Tappe et al., 2014; Saiz et al., 2016; Venturi et al., 2016).

Continuous global surveillance is advised as situations may change following the start of warmer weathers, allowing ZIKV mosquito vectors to become active (Imperato, 2016; Saiz et al., 2016).

### Molecular classification

**Figure 1:**
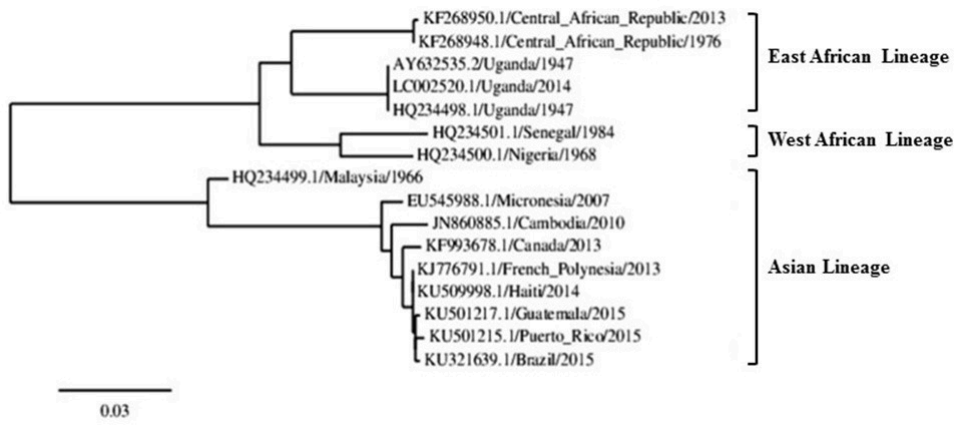
**ZIKV phylogenetic tree showing the three ZIKV lineages constructed using the complete NS5 nucleotide sequence**. The scale bar indicates 0.03 substitutions/site. Modified from: Saiz et al. (2016).

Although, limited molecular data of the ZIKV genome sequence from human isolates are currently available, sufficient data exists to determine the evolutionary patterns of the virus (Petersen L. R. et al., 2016). Through ZIKV genome sequencing and phylogenetic analysis of several human isolates, three geographically distinct lineages of ZIKV have been reported, including the East African, West African, and the Asian strains (see Figure 1). It is postulated that ZIKV originated in the Eastern region of Africa and then spread toward the West (Lanciotti et al., 2016). In the late 1960s, the first Asian ZIKV was isolated in Malaysia and then subsequently, the virus spread across southeast Asia (Marchette et al., 1969). It is postulated that the Asian ZIKV strain emerged as a result of accumulation of mutations in the ZIKV genome, hence, introducing new molecular interacting partners with the host cell factors and changes in disease pathogenicity, vector competence, and epidemic potential (Lazear and Diamond, 2016; Wang et al., 2016; Weaver et al., 2016). At this point, it must be noted that when compared to other RNA viruses, fixation of a mutation in the genome of an arbovirus faces greater constraints due to the presence of important genes required for replication in mammals and invertebrates (Holmes, 2003; Hamel et al., 2015).

The current ZIKV outbreaks in the Americas, and previously in the French Polynesia and Yap Islands, are reported to be most closely related to the Asian strain (see Figure 1; Haddow et al., 2012; Imperato, 2016). Through genome analysis and phylogenetic studies, it was found that the ZIKV currently circulating in the Americas share >99% identity with ZIKV isolates from the French Polynesia outbreak and is 89% identical to the African strain (Lanciotti et al., 2016; Lazear and Diamond, 2016). The high genomic similarity of the ZIKV strains circulating in the Americas allow for targeted drug and therapeutic development (Petersen L. R. et al., 2016). Recent studies focusing on the phylogenetic relationship between ZIKV and other *Flaviviruses* have also contributed to the understanding of the nature of ZIKV. Upon constructing phylogenetic trees based on the non-structural viral protein 5 (NS5) and the structural viral protein E, it was found that although the ZIKV has the potential to affect the central nervous system, particularly in neonates, it however is not particularly related to other encephalitic viruses (Wong et al., 2016).

### Virology and pathogenesis

**Figure 2:**
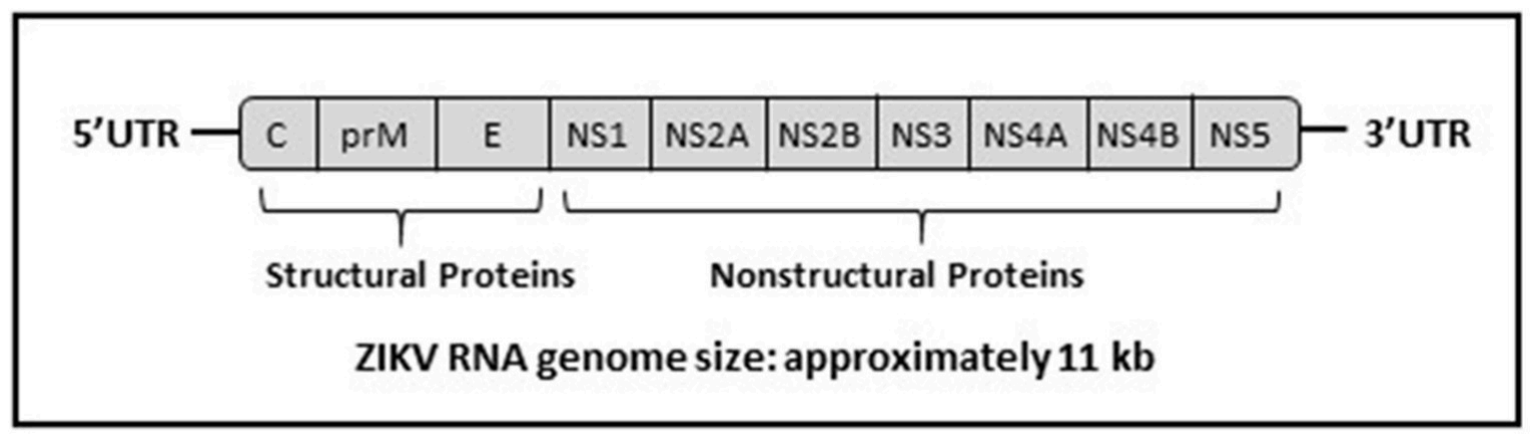
**Genomic structure of ZIKV flanked by the 3′ and 5′ untranslated region (UTR)**. Redrawn from: Marano et al. (2016).

ZIKV, a biosafety level-2 pathogen, has an enveloped positive-sense, single-stranded RNA genome with a size of approximately 10,676 bp and is known to be closely related to the Spondweni virus (Charrel et al., 2016; Wong et al., 2016). ZIKV virions are approximately 60 nm in size and spherical in shape (Charrel et al., 2016). The ZIKV genome encodes for a single polyprotein (approximately 3400 amino acids) that is subsequently processed by host and viral proteases into ten different proteins, consisting of three structural and seven nonstructural proteins (see Figure 2; Saiz et al., 2016). Table 1 summarizes the function of each ZIKV protein based on the general information collectively available for *Flaviviruses* (Wong et al., 2016).

**Table 1:** **Function and cellular localization of ZIKV proteins (Kostyuchenko et al., 2016; Lazear and Diamond, 2016; Wong et al., 2016)**.

**Viral proteins**	**Protein localization**	**Function**
E	Structural surface protein	Host receptor binding, host cell fusion, and viral entry
prM, M	Structural surface protein	E protein stabilization and host cell fusion
C	Structural core protein	Binds to viral RNA for nucleocapsid formation
NS1		Viral replication
NS2a		Viral transcription and assembly
NS2b		NS3 cofactor for appropriate serine protease function
NS3	Not a part of the virus particle. Encoded by the viral RNA genome and translated using host cellular machinery	Serine protease, RNA helicase, and triphosphatase activity
NS4a		Viral replication
NS4b		Viral replication
NS5		Viral replication, RNA-dependent RNA polymerase, RNA capping, methyltransferase activity

Currently, little information is available regarding ZIKV pathogenesis, as compared to other members of the *Flavivirus* genus. Generally, arboviruses (mosquito-borne viruses) are known to replicate in dendritic cells and subsequently disseminate to the lymph nodes and bloodstream (Diamond et al., 2004). Within the cells, replication of *Flaviviruses* is known to occur in the cytoplasmic region however, ZIKV antigens have been observed within the cell nuclei. Hence, it is suggested that ZIKV replication may differ from that of other *Flaviviruses* (Buckley and Gould, 1988). In addition, according to a study by Priyamvada et al. (2016), the potential association between ZIKV and prenatal microcephaly/GBS may be attributed by the introduction of ZIKV into a population with high flaviviral background (e.g., prior exposure to DENV). The immunity established against other *Flaviviruses* may play a role in the modulation of ZIKV pathogenesis (Fajardo et al., 2016).

To study ZIKV pathogenesis, efforts have been put toward the investigation of murine models with different manifestations of ZIKV infection (Shah and Kumar, 2016). A study by Dick (1952) showed motor weakness and paralysis in mice intracerebrally infected with ZIKV strain M766 isolated from the brain of young infected mice. Previous studies have also suggested that glycosylation of viral E protein is associated with the ability of *Flaviviruses* to cause a pandemic (Shirato et al., 2004). Recently, E protein analysis of multiple pathogenic ZIKV strains circulating in the Americas indicated positive glycosylation patterns. In contrast, majority of the other ZIKV strains were found to lack E protein glycosylation (Baronti et al., 2014; Berthet et al., 2014). These findings suggest that E protein glycosylation may be indicative of ZIKV pathogenicity (Saiz et al., 2016).

### Host cell-virus interaction

According to a study by Hamel et al. (2015), ZIKV host cell entry and endocytosis occurs via interactions of viral E proteins with host cell adhesion factors, such as DC-SIGN (Dendritic Cell-Specific Intercellular adhesion molecule-3-Grabbing Non-integrin) and multiple members of the phosphatidylserine receptor family. The acidic environment within the endosome promotes viral envelope and endosome membrane fusion (low pH promotes E glycoprotein rearrangement) hence, allowing the release of ZIKV RNA genome into the cytoplasm for the initiation of translation (Saiz et al., 2016). Subsequently, translated viral proteins aid in viral genome replication at the surface of the endoplasmic reticulum (ER) (Hamel et al., 2015). Within the ER, positive strand viral RNA is packaged to form immature virions. The virus matures in the trans-Golgi network, upon the cleavage of prM into M protein. ZIKV is then released into the surroundings via exocytosis (Roby et al., 2015). Scarce information is available regarding host cell response to viral genome replication. According to Hamel et al. (2015), ZIKV replication induces innate viral response and transcription of interferon stimulated genes.

### Symptoms

Up to 80% of human ZIKV infections appear to be asymptomatic, with a small subset of cases presenting with mild clinical symptoms similar to other flaviviral and influenza infections (Marano et al., 2016; Shah and Kumar, 2016). Clinical manifestation in symptomatic cases tend to appear after an incubation period of 3 to 12 days and are reported to be characterized by fever, rashes, myalgia, arthralgia, conjunctivitis, gastrointestinal disturbance, and headaches (Buathong et al., 2015; Basarab et al., 2016). However, major concern is associated with the steep increase in reported cases of prenatal microcephaly and GBS in the Americas after the recent ZIKV outbreak (Mlakar et al., 2016). To this regard, it must be pointed out that prenatal microcephaly and GBS have been implicated in other flaviviral infections, such as by DENV and WNV (Weaver et al., 2016). Teratogenic effects of flaviviral infections have been reported to target the eyes and brain. Recent studies have suggested ZIKV infections to be highly neurotrophic, with a few cases reporting association with bilateral macular and perimacular lesions (Mlakar et al., 2016; Ventura et al., 2016). Such complications following ZIKV infection were undocumented in the 1950s. Hence, it is evident that ZIKV genetic evolution (emergence of Asian lineage) has resulted in increased virus pathogenicity (Oehler et al., 2014; Rasmussen et al., 2016).

In 2015, amid the ZIKV epidemic, an astounding 20-fold increase in prenatal microcephaly cases were reported in Brazil, as compared to 2014 (Fauci and Morens, 2016). Since October 2015 up until now, approximately 4000 ZIKV infection-related prenatal microcephaly cases have been reported in Brazil, causing over 40 infant deaths (Higgs, 2016). During the outbreak, Paraíba (north-east Brazil), a ZIKV endemic state, was reported to have significantly increased cases of prenatal microcephaly, from 5.7 per 100,000 live births in 2010 to 99.7 per 100,000 live births (Basarab et al., 2016). In addition, 1708 cases of GBS were reported in 2015 in Brazil, a 19% increase as compared to the preceding year (Basarab et al., 2016). Besides that, a case-control study based on the French Polynesia ZIKV outbreak indicated that patients with GBS were more likely to have a history of ZIKV as compared to the control group (Cao-Lormeau et al., 2016). Another study reported 20-fold increase in GBS cases following the French Polynesia outbreak (Oehler et al., 2014).

A recent study on mouse models reported teratogenic effects, such as neuronal cell death and microcephaly, in pups born to SJL mice infected with ZIKV during pregnancy (Cugola et al., 2016). The precise mechanism by which ZIKV causes prenatal microcephaly or GBS is yet unknown. Increased incidences of microcephaly and GBS in regions positive for ZIKV circulation and evidence from clinical and epidemiological studies have increasingly been pointing toward a plausible causal association (Rasmussen et al., 2016). Table 2 summarizes the studies supporting the plausible association between ZIKV infection and prenatal microcephaly and GBS.

**Table 2:** **Summary of reported association between ZIKV infection and prenatal microcephaly (PM) / GBS**.

**Country**	**Year**	**No. of patients**	**Symptom**	**Sample tested**	**ZIKV detection method**	**References**
Slovenia	2015	1	PM	Fetal brain tissue	qRT-PCR	Mlakar et al., 2016
Hawaii	2015	1	PM	Not Reported	Laboratory confirmation (method not described)	Hawaii Department of Health, 2016
Brazil	2015	2	PM	Fetal brain tissue	RT-PCR & Anti-ZIKV ELISA	Martines et al., 2016
Brazil	2015	2	PM	Amniotic fluid	qRT-PCR & ZIKV sequencing	Calvet et al., 2016
Brazil	2015	1	PM	Fetal blood & tissue	Genome detection (method not described)	Pan American Health Organization, 2016a
Brazil	2015	2	Miscarriage	Placental tissue	RT-PCR & Anti-ZIKV ELISA	Martines et al., 2016
Brazil	2015	1	PM	Fetal cerebral cortex, medulla oblongata, cerebrospinal, & amniotic fluid	qPCR	Sarno et al., 2016
Brazil	2015	12	PM	Fetal cerebrospinal fluid	Anti-ZIKV IgM ELISA	Lazear and Diamond, 2016
Brazil	2015–2016	12	Fetal Abnormalities	Maternal urine and/or blood	qRT-PCR	Brasil et al., 2016
Brazil	2016	1	PM	Fetal brain tissue, membranes, placenta, & umbilical cord	qRT-PCR	Driggers et al., 2016
French Polynesia	2013	1	GBS	Serum	Anti-ZIKV IgG ELISA & PRNT	Oehler et al., 2014
French Polynesia	2013–2014	42	GBS	Serum	Seroneutralization assay & Anti-ZIKV IgM/IgG ELISA	Cao-Lormeau et al., 2016
Martinique	2015	2	GBS	Urine	RT-PCR	World Health Organization, 2016a
Brazil	2015	7	GBS	Not Reported	Lab Confirmed	World Health Organization, 2016c
Brazil	2015	4	GBS	Serum or cerebrospinal fluid	RT-PCR and/or Anti-ZIKV IgM ELISA	Araujo et al., 2016
Suriname	2015	2	GBS	Not reported	RT-PCR	World Health Organization, 2016c
Puerto Rico	2015–2016	5	GBS	Serum	RT-PCR and/or Anti-ZIKV IgM ELISA	Dirilikov et al., 2016
Venezuela	2016	6	GBS	Not Reported	RT-PCR	World Health Organization, 2016b
Venezuela	2016	1	GBS	Serum & cerebrospinal fluid	qRT-PCR	Mécharles et al., 2016

*PM, Prenatal microcephaly; GBS, Guillain-Barré syndrome; RT-PCR, reverse transcription polymerase chain reaction; qRT-PCR, real time quantitative RT-PCR; Ig, Immunoglobulin; ELISA, Enzyme-Linked Immunosorbent Assay*.

### Life cycle

**Figure 3:**
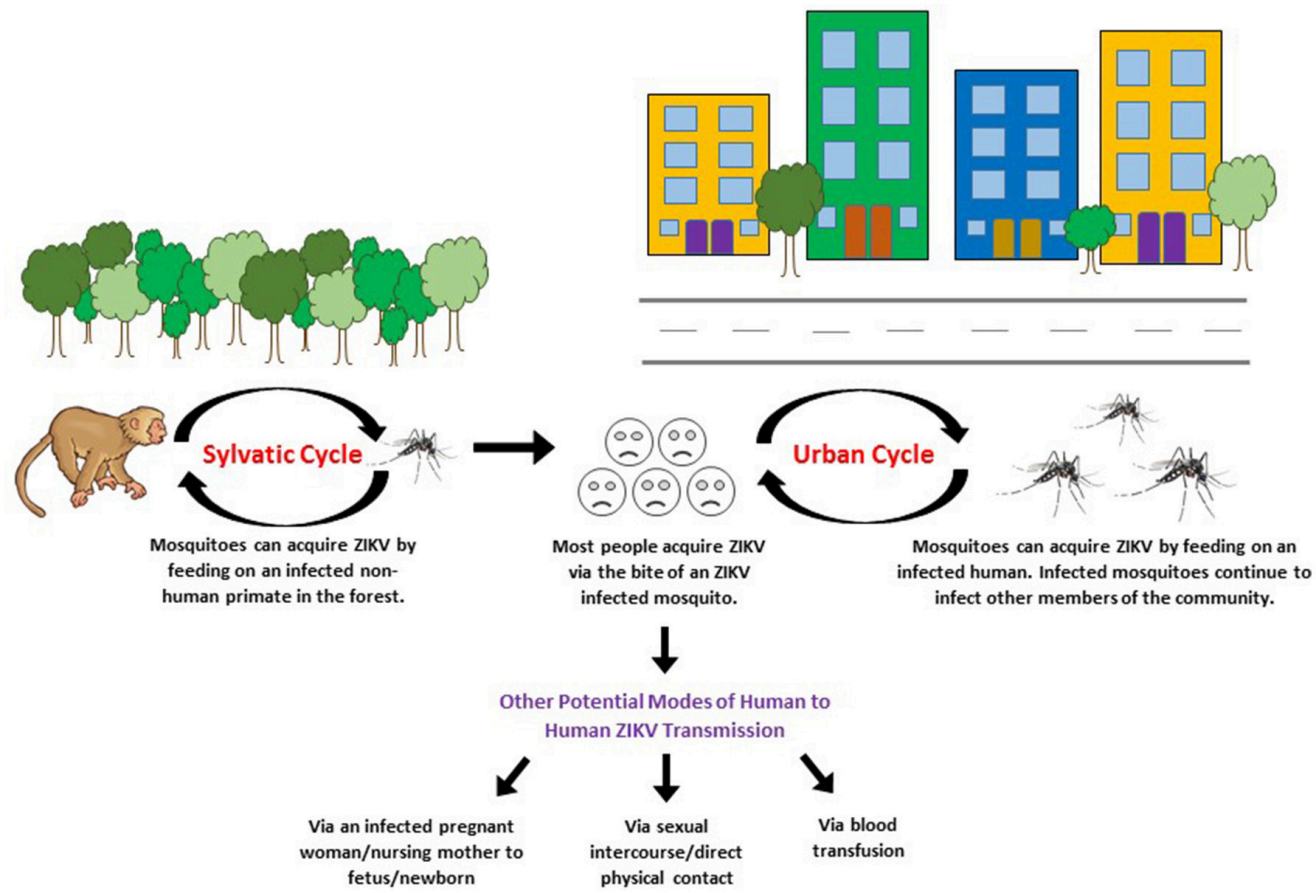
**Sylvatic/enzootic and urban transmission cycle of ZIKV**. Redrawn based on the information obtained from: Besnard et al. (2014), Musso et al. (2014), Musso et al. (2015b), Swaminathan et al. (2016), Weaver et al. (2016).

To date, ZIKV has been isolated from a vast range of organisms, including humans, non-human primates (apes, monkeys, and orangutans), and mosquitoes. Antibodies against ZIKV have also been detected in vertebrates (rodents, birds, sheep, goats, cattle, reptiles), hence, suggesting their potential role in the circulation of ZIKV (Johnson et al., 1977). The African ZIKV lineage is thought to be maintained via the sylvatic/enzootic transmission cycle primarily between non-human primates (apes and monkeys) and mosquitoes, with humans as incidental hosts. However, humans have most likely become the prominent host for the Asian ZIKV lineage (Althouse et al., 2016; Basarab et al., 2016). Through evolution, ZIKV has gained the ability to sustain transmission in a human-endemic cycle (suburban-urban transmission cycle) thus, allowing humans to serve as the carrier, multiplier, and source of ZIKV for uninfected mosquitoes (see Figure 3; Saiz et al., 2016). The suburban-urban transmission cycle is thought to cause and sustain epidemics (Lazear and Diamond, 2016). Current research on ZIKV life cycle focuses on determining the possibility and impact of ZIKV sylvatic cycle establishment within the Americas. Such studies have highlighted the importance of targeted surveillance of the susceptible animal population for enzootic ZIKV (Fauci and Morens, 2016).

## Zikv transmission dynamics

### Mosquito-borne transmission

ZIKV transmission to humans occur primarily through bites of an infected, day-dwelling female *Aedes aegypti* or *Aedes albopictus* mosquito, similar to the transmission of chikungunya virus (CHIKV) and DENV. *A. aegypti* mosquitoes are confined to the tropical and sub-tropical regions, hence, limiting ZIKV transmission potential (Petersen L. R. et al., 2016). However, *A. albopictus* mosquitoes are known to be geographically distributed throughout the tropical, subtropical, and temperate regions, hence, allowing for greater transmission potential (Thomas et al., 2012). The exact incubation period for ZIKV before the mosquito becomes capable of transmitting the virus is yet unknown. However, according to Hayes (2009), the extrinsic incubation period of ZIKV in mosquitoes is suggested to be approximately 10 days. Currently, it is presumed that uninfected mosquitoes are capable of acquiring ZIKV by feeding on an infected human (approximately during the time of clinical manifestation in humans). To this regard, further studies must be conducted to confirm that the viral titer level in the serum of infected individuals is sufficient to infect a naïve mosquito (Grard et al., 2014).

To date, ZIKV has been isolated from 17 different *Aedes* mosquito species as well as *Culex perfuscus, Mansonia uniformis, Anopheles coustani*, and *Anopheles gambiae* mosquitoes (Ayres, 2016; Saiz et al., 2016; Slavov et al., 2016). It is postulated that the enzootic maintenance of ZIKV occurs through these mosquito species however, ZIKV transmission to humans is contributed only by a subset of these species (Lazear and Diamond, 2016). To date, vector competence and maintenance of the suburban-urban ZIKV transmission has been reported in *A. aegypti, A. albopictus, A. hensilli* (responsible for Yap Island outbreak), and *A. polynesiensis* (responsible for French Polynesia outbreak) (Imperato, 2016; Lazear and Diamond, 2016). The principle vector currently responsible for spreading ZIKV within the Americas include the *A. aegypti* and *A. albopictus* species (Petersen L. R. et al., 2016). The involvement of a diverse range of mosquito species in the maintenance of ZIKV suggests that the transmission dynamics of ZIKV is complex (Althouse et al., 2016).

### Sexual transmission

Multiple cases of male-to-female ZIKV transmission have been reported thus, raising the concern of a novel mode of ZIKV transmission in the human semen (Imperato, 2016). In 2011, a case study reported ZIKV transmission from an infected male to his female partner via sexual intercourse after the patient returned from Senegal to the United States (Foy et al., 2011). Serologic testing detected ZIKV RNA in both male and female partners. A similar study indicated the development of ZIKV infection in a female (confirmed via RT-PCR on serum sample) 13–14 days after having sexual intercourse with an infected male who had recently returned from the Caribbean (Hills et al., 2016). Three other similar cases have also reported coherent findings (Foy et al., 2011; Hills et al., 2016). In all the cases reported, the female partners had not traveled out of the United States and local mosquito-borne transmission was not considered due to vector absence within the geographical location. Until recently, ZIKV has been thought to be transmitted only from males to their sexual partner. On 15 July 2016, the first female-to-male sexual transmission of ZIKV was reported, further raising concerns that ZIKV could spread more widely (Centers for Disease Control and Prevention Newsroom, 2016; Davidson et al., 2016; Santora, 2016).

Recently, a study reported detection of ZIKV RNA via qRT-PCR in the semen of infected males up to 188 days after the onset of symptoms, even after viremia had cleared (serum negative for ZIKV RNA) (Nicastri et al., 2016). The detection of high infectious viral load and ZIKV RNA in semen suggest prolonged potential for sexual transmission (Atkinson et al., 2016; Mansuy et al., 2016; Nicastri et al., 2016). However, the mechanism underlying the sexual transmission of ZIKV from a male to the female partner is yet unknown. ZIKV is by far the first arbovirus to be detected in human semen (Musso et al., 2015b).

Recent studies have reported the detection of ZIKV RNA and infectious viral load in the saliva and urine of infected individuals (Gourinat et al., 2015; Barzon et al., 2016). Distinguishing between sexual and salivary/urinary transmission of ZIKV becomes difficult due to the correlated nature of behavior associated with sexual activity (Foy et al., 2011; Musso et al., 2015a). Verification of ZIKV transmission via sexual interaction could significantly change the epidemiology of ZIKV as ZIKV RNA was found to be detectable in semen over a longer period of time, as compared to blood serum (Foy et al., 2011; Atkinson et al., 2016).

### Blood transfusion-related transmission

During the French Polynesia outbreak, ZIKV RNA was detected in approximately 3% of asymptomatic blood donors (acute phase of infection) thus, making blood transfusion a novel potential mode of ZIKV transmission (Musso et al., 2014; Basarab et al., 2016). ZIKV transmission via blood transfusion is plausible as ZIKV infections are primarily asymptomatic and blood transfusion-related transmission of other *Flaviviruses* have been reported (Marano et al., 2016; Shah and Kumar, 2016). The first confirmed case of blood transfusion-related ZIKV transmission has been recently reported in Brazil (Centers for Infectious Disease Research and Policy, 2016). To address this issue, on 19 February 2016, the WHO issued strict guidelines for blood transfusion/donation in regions where ZIKV was endemic (Imperato, 2016). In multiple countries, such as in Europe, United States, and Canada, donated blood is screened via nucleic acid testing to detect WNV RNA (O'Brien et al., 2010; Centers for Disease Control and Prevention, 2013; Pupella et al., 2013). Adopting the same approach, continuous efforts are in place to formulate a simple yet precise test to detect ZIKV in donated blood. An alternative option is to avoid blood donation from individuals within ZIKV endemic regions or with recent history of travel to those regions (Lazear and Diamond, 2016). To further improve transfusion safety, pathogen reduction technologies are also being utilized to render pathogens inactive (Marano et al., 2016). Nevertheless, further studies need to be conducted to detect ZIKV and its transmission in donated blood (Saiz et al., 2016).

### Maternal transmission

#### Prenatal transmission

ZIKV has reportedly been detected in microcephalic neonates born to mothers with a history of ZIKV infection during pregnancy (Besnard et al., 2014; Centers for Disease Control and Prevention, 2016a). It is postulated that ZIKV has the ability to cross the placenta and subsequently, infect fetal nervous tissues. The suggested mechanism is supported by the evident detection of ZIKV RNA and antigens in the amniotic fluid, placenta, and fetal brain tissue as well as visualization of ZIKV particles in fetal brain via electron microscopy (Calvet et al., 2016; Lazear and Diamond, 2016; Petersen L. R. et al., 2016). It is a known fact that the placenta acts as an effective immunological barrier between the mother and the fetus, protecting the fetus from microorganisms in the mother's circulation. The mechanism used by ZIKV to circumvent the placental barrier is yet to be discovered (Bayer et al., 2016). A recent study on mouse models discovered that ZIKV infection during pregnancy resulted in placental damage and fetal death, further supporting the trans-placental route of transmission (Miner et al., 2016). The potential of ZIKV to undergo *utero* transmission has raised global concerns as regions positive for ZIKV circulation, such as Brazil, have recently reported a tremendous increase in the cases of prenatal microcephaly. Accumulating evidences, as shown in Table 1, suggest the potential ability of ZIKV to be transmitted to the fetus and the potential role of ZIKV in the development of prenatal microcephaly (Lazear and Diamond, 2016).

#### Nursing mothers

ZIKV RNA and infectious viral particles have been detected in high loads in the breast milk of infected mothers (Dupont-Rouzeyrol et al., 2016). This introduces a novel transmission mechanism in which ZIKV transmission occurs from the mother to the nursing child. According to a mother-infant pair study, ZIKV RNA was detected in the breast milk and serum of two mothers and in the serum of their respective infants (Besnard et al., 2014). Particularly, serum sample from one of the infants tested positive via RT-PCR after breastfeeding. However, ZIKV replication was not detected upon inoculation of the breast milk on Vero cells hence, making transmission via breast milk uncertain yet plausible. Other potential confounding mother-to-child ZIKV transmission routes should be further investigated. *Flavivirus* transmission, such as DENV and WNV, via breast milk have been previously reported (Ognjan et al., 2002; Barthel et al., 2013).

### Transmission by physical contact

In September 2016, the first case of ZIKV transmission via direct physical contact was reported in the United States, further suggesting a sophisticated and complex ZIKV transmission mechanism (Swaminathan et al., 2016). The study reported the transmission of ZIKV from an infected patient (Patient 1: 73-year-old) to his healthy son (Patient 2: 38-year-old). Patient 1 had returned to the United States from the southwest coast of Mexico, where ZIKV transmission had been recorded, 8 days prior to hospitalization in Salt Lake City. Patient 1's serum assay for ZIKV via real-time PCR was positive, with an estimation of a very high viral load. In addition, high-throughput RNA sequencing of the ZIKV isolated from Patient 1 revealed 99.8% similarity to the genome sequence of a ZIKV strain circulating in mosquitoes in Chiapas, Mexico, in 2016. Patient 1 died 4 days after hospitalization. Subsequently, 5 days after Patient 1's death, Patient 2 developed ZIKV symptoms. On day seven post-symptom onset, Patient 2's urinalysis via PCR assay and serum IgM antibody test were positive for ZIKV, although blood serum analysis for ZIKV via PCR was negative. Patient 2 had visited Patient 1 during hospitalization and reported to have wiped Patient 1's watering eyes without gloves and assisted a nurse in repositioning Patient 1. None of the health care workers who had contact with the patients reported having symptomatic illness. Since the *Aedes* mosquito species known to transmit ZIKV are absent in the Salt Lake City area and Patient 2 had not recently traveled to a ZIKV endemic region and had not had sex with a partner with recent travel history to such areas, it is most likely that Patient 2 acquired the ZIKV infection from Patient 1, whose sweat or tears may have contained infectious ZIKV (Swaminathan et al., 2016).

## Modes of detection

**Figure 4:**
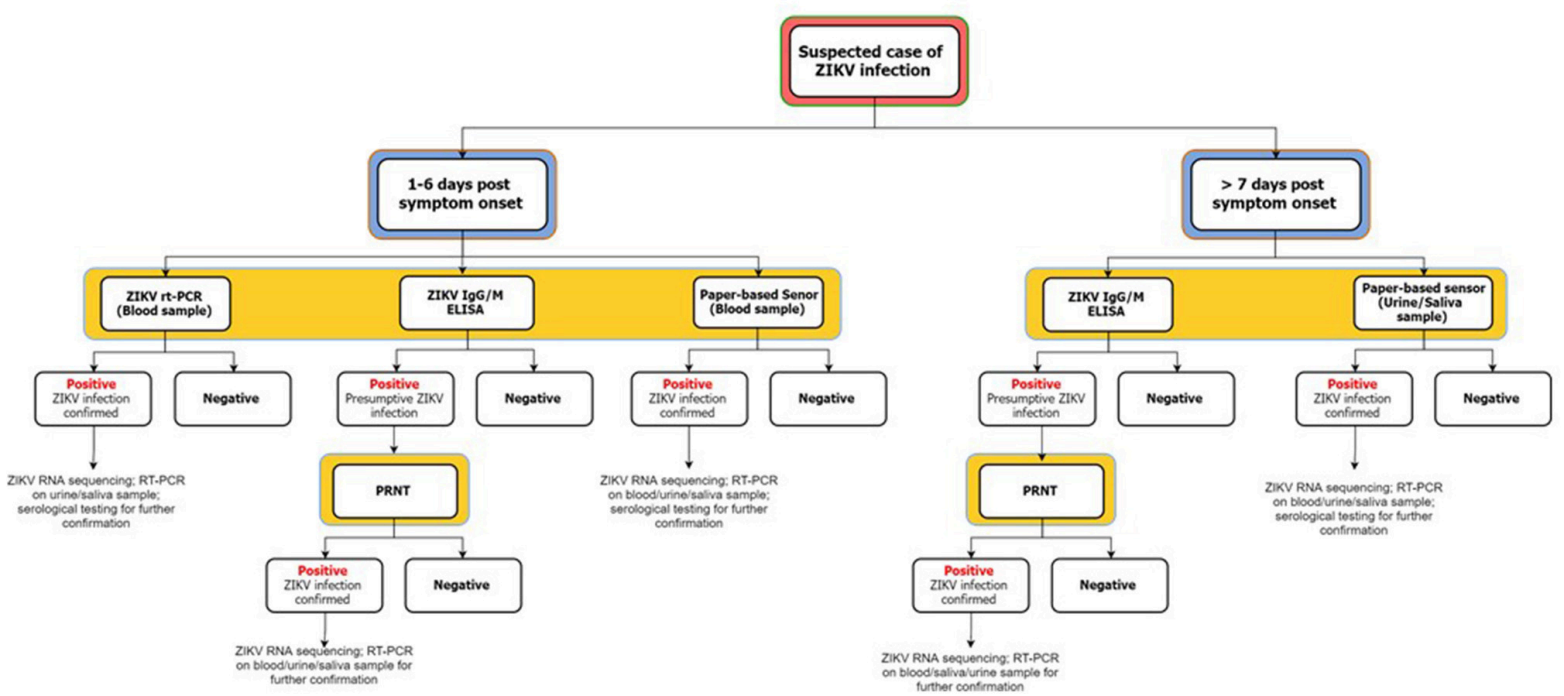
**Proposed flowchart for ZIKV infection diagnosis**. This figure is self-drawn based on the information provided in the text (Summary of Section: Modes of Detection).

The recent ZIKV outbreak in the Americas and its continuous spread, along with increased likelihood of causal association with prenatal microcephaly and GBS has prompted a search for a low-cost and rapid ZIKV detection method (Pardee et al., 2016; Vorou, 2016). The present ZIKV outbreaks have been reported to be of the Asian lineage, hence, current research focuses on developing Asian strain-specific detection assays (Charrel et al., 2016). As mentioned earlier, scarce information is available regarding the pathogenesis of ZIKV hence, understanding of the ZIKV transmission dynamics could potentially aid in the development of a robust detection technique. To date, standardized tests for ZIKV detection have not yet been developed (Fauci and Morens, 2016). In addition, clinical presentations of ZIKV infection appear to be highly similar to other arboviral infections, such as DENV and CHIKV infection, hence, potentially confounding diagnosis (Basarab et al., 2016; Fauci and Morens, 2016). In 2015, 224 dengue patients were screened for ZIKV infection, with seven out of 10 individuals testing positive for ZIKV infection (Agencia Fiocruz de Noticias, 2016). However, it must be noted that ZIKV diagnosis and confirmation is challenging due to cross-reactivity and low viremia (Gourinat et al., 2015). During ZIKV testing, cross-reactivity to other *Flaviviruses* often occurs due to close-relatedness and co-circulation of other *Flaviviruses* in ZIKV endemic regions (ZIKV infection is secondary) (Basarab et al., 2016; Charrel et al., 2016). Detection of ZIKV is best during the acute-phase, however, it is difficult to determine the period for onset of symptoms as majority of the cases are asymptomatic (Shah and Kumar, 2016). Figure 4 summarizes multiple techniques for ZIKV infection diagnosis.

### Reverse transcription polymerase chain reaction (RT-PCR)-based detection

Real-time and conventional RT-PCR are the most common approaches utilized in diagnostic labs owing to their specificity and ability to differentiate ZIKV from other flaviviral infections (Wong et al., 2016). RT-PCR allows for rapid, specific, and reliable ZIKV RNA detection during the acute-phase, as compared to other modes of detection (Marano et al., 2016). The development of ZIKV specific primers for nested RT-PCR has been reported to increase specificity (Grard et al., 2014). Specific RT-PCR molecular assays have been developed for the detection of Asian and African ZIKV strains. Often, the ZIKV envelope genes (prM/E protein coding regions) are targeted for amplification due to their unique characteristics which allow for differentiation from other *Flaviviruses* (Musso et al., 2015b; Marano et al., 2016). Two specific sets of primers for the Asian ZIKV strain have been tested and established (Lanciotti et al., 2008). To further increase specificity, the use of TaqMan probe is recommended (Charrel et al., 2016). Commercial kits (for research purposes) for ZIKV RNA detection via RT-PCR have recently entered the market (Charrel et al., 2016).

Peripheral blood samples are predominantly used for PCR-based assays (Wong et al., 2016). However, RT-PCR on blood and serum samples is associated with reduced sensitivity due to low viremia in humans (Vorou, 2016). More recently, detection of higher viral RNA load over a longer duration was reported in urine and semen samples (Musso et al., 2015a,b). Related studies have coherently reported detection of higher DENV and WNV RNA load over a longer duration in urine samples, as compared to blood serum (Musso et al., 2015b). ZIKV RNA has also been reported to be detected in the saliva of infected individuals, often more readily compared to blood samples (Musso et al., 2015a). The choice and combination of samples chosen for testing is highly dependent on the stage of infection (see Table 3). It is recommended to perform RT-PCR on both blood and saliva/urine samples in order to increase test sensitivity, particularly during the late stage of infection (Gourinat et al., 2015; Musso et al., 2015a). In addition, alternative sampling of urine or saliva reduces invasiveness and hence, is advantageous for diagnosis in neonates and infants (Charrel et al., 2016). For prenatal testing, amniotic fluid is predominantly collected for molecular analysis. A positive RT-PCR for ZIKV RNA is suggestive of intrauterine infection and plausible reduction in fetus fitness (Petersen E. E. et al., 2016). Ultimately, products of ZIKV RNA specific RT-PCR amplification, regardless of sample source, cam also be sequenced and aligned against established ZIKV genome sequences for confirmation (Musso et al., 2015b).

**Table 3:** **Molecular detection of ZIKV RNA from different human sample types**.

**Sample type**	**Primary detection technique**	**Duration for detection**	**References**
**Blood**	qRT-PCR	Within approximately 5–7 days after onset of symptoms	Vorou, 2016
**Serum**	qRT-PCR & Antibody-based detection	Within approximately 5–6 days after onset of symptoms	Pan American Health Organization, 2016a
**Semen**	qRT-PCR	ZIKV RNA detected up to 62 days after onset of symptoms	Atkinson et al., 2016
**Urine**	qRT-PCR	Within approximately 15 days after onset of symptoms	Gourinat et al., 2015
**Saliva**	qRT-PCR	ZIKV RNA detected up to 20 days after onset of symptoms	Musso et al., 2015a

### Antibody-based detection

#### Immunoglobulin (Ig) G/M enzyme-linked immunosorbent assay (ELISA)

IgM/IgG ELISA involves the detection of ZIKV-specific antibodies in the serum (Huzly et al., 2016). IgM antibodies are known to develop within a few days post onset of symptoms and can last up to 3 months. IgG antibodies on the other hand, develop after IgM and can last from a few months to years. IgM specific to ZIKV have been developed at the Centers for Disease Control and Prevention, Atlanta (Hayes, 2009). However, studies have reported complications during diagnosis due to sera cross-reactivity of ZIKV IgM to antibodies against other *Flaviviruses*, often in patients with a history of flaviviral infection or vaccination (Charrel et al., 2016). According to Hayes (2009), cross-reactivity was predominantly noted with DENV, as compared to other *Flaviviruses*. IgM against ZIKV was detected in the serum as early as 3 days post onset of symptoms. However, in certain cases, IgM was detected after the 8th day post-symptom onset, thus introducing uncertainties in diagnosis. Commercial kits (for research purposes) for rapid ZIKV IgM/IgG ELISA detection are readily available in the market (Charrel et al., 2016).

#### Plaque reduction neutralization test (PRNT)

PRNT is used for virus-specific neutralizing antibody titer quantification (Rabe et al., 2016). The test is reported to have improved specificity compared to ELISA hence, it is often used in addition to ELISA to rule-out false positive antibody response (Hayes, 2009; Oehler et al., 2014; Charrel et al., 2016). The PRNT was used in addition to ELISA for diagnosis and confirmation of ZIKV in 185 patients during the French Polynesia outbreak (Duffy et al., 2009). To perform the PRNT, firstly, serum sample from the patient was diluted and mixed with a suspension of ZIKV. Subsequently, the mixture was poured over a monolayer of cells, often Vero or LLC-MK2 cell lines (Lednicky et al., 2016). The cells were subsequently covered with a thin layer of agar to avoid viral movement. PRNT against other *Flaviviruses* are concurrently performed as a control (Rabe et al., 2016). At least a 4-fold increase in ZIKV-specific neutralizing antibody titer is recommended for confirmation of ZIKV infection (Pan American Health Organization, 2016a). However, interpretation of results could be complicated if high *Flavivirus* background is observed in the patient, often due to history of vaccination against *Flaviviruses* (Lanciotti et al., 2008; Rabe et al., 2016).

### Toehold switch sensor and CRISPR/cas9-based detection

A novel, rapid, and low-cost method for ZIKV RNA detection has recently been suggested by Pardee et al. (2016). The study introduces a pipeline for ease of ZIKV RNA detection and ZIKV strain differentiation using a cell-free approach. Firstly, an RNA sensor, also known as the toehold switch sensor, programmable to bind and detect essentially any specific RNA sequence was developed. For ZIKV detection, the sensors were designed to bind specifically to ZIKV RNA and become activated at concentrations as low as 30 nM. Subsequently, the sensors were embedded into paper and freeze-dried. This increased sensor stability and ease of distribution to ZIKV endemic regions. The simple design of the paper-based sensor allows for rapid mass production at a cost as low as US$1 per sensor (Dockrill, 2016).

For detection using the paper-based sensor, total RNA must first be isolated from blood, serum, saliva, or urine and then subjected to ZIKV genome region specific amplification using the nucleic acid sequence-based amplification (NASBA) technique in order to boost ZIKV RNA signaling (Pardee et al., 2016). Subsequently, the amplified product is to be applied to the ZIKV RNA-specific sensor. Samples positive for ZIKV RNA are visually distinguishable upon the change of sensor color (from yellow to purple). The viral load in the sample could also be determined via semi-quantitative analysis (as described in Pardee et al., 2016). Recently, a CRISPR/Cas9 based module has also been coupled to the NASBA system hence, allowing different ZIKV strains to be distinguished with single-base resolution (Pardee et al., 2016). Due to the high specificity of the sensor to the targeted ZIKV genomic region, cross-reactivity to other closely related *Flaviviruses* has been eliminated. Serum, urine, and saliva samples from infected patients have been tested using the novel innovation. ZIKV RNA was successfully documented, with higher loads reported in urine samples as compared to serum and saliva (Pardee et al., 2016).

## Control measures

### Vaccine development

Vaccines have been successfully developed for protection against multiple *Flaviviruses*, such as YFV, TBEV, JEV, and DENV (Lazear and Diamond, 2016; Weaver et al., 2016). To date, no vaccine against ZIKV has entered the clinical stage, therefore, suggesting that ZIKV vaccine establishment is yet multiple years away (Lazear and Diamond, 2016). Recently, an Indian biotech company claimed that it has two ZIKV vaccine candidates awaiting pre-clinical trials (Macdonald, 2016). SynCon Pharmaceuticals (USA) has also developed a DNA-based vaccine against ZIKV, which is expected to enter clinical trials by the end of this year (Saiz et al., 2016). Current ZIKV vaccine development strategies have been targeted toward adaptation of existing *Flavivirus* vaccine platforms (e.g., inactivated or live-attenuated virus, *Flavivirus* chimera, glycoprotein subunit technology). The growing threat of an explosive global spread of ZIKV has drawn an alarming interest among the scientific community to develop a suitable murine model for vaccine development (Shah and Kumar, 2016). Since low genetic variation is observed in different ZIKV strains, it is likely that a single vaccine may be effective against all circulating ZIKV strains. However, the effect of pre-existing immunity against other *Flaviviruses* on the immunity against ZIKV must be further investigated (Lazear and Diamond, 2016). In addition, it must be noted that *Flavivirus* vaccine development is limited by the nature of outbreaks, being sporadic and unpredictable. Therefore, rapid vaccine production to counter the quick spread of ZIKV may pose difficulties. Besides that, preemptive vaccination in anticipation of an outbreak may appear to be prohibitively expensive (Fauci and Morens, 2016).

### Antiviral therapeutics

Since ZIKV vaccine clinical trials are yet underway, more efforts are being put toward developing antiviral therapeutics against ZIKV for immediate control (Lazear and Diamond, 2016). Hitherto, there is no established antiviral treatment available for flaviviral infections (Weaver et al., 2016). Current ZIKV infection treatment is symptomatic, often through the use of analgesics and antipyretics (Petersen E. E. et al., 2016). Human neural progenitor cells derived from induced pluripotent stem cells have been developed for use as an *in vitro* platform for therapeutic compound screening (Tang et al., 2016). Often, drugs with inhibitory activity against a specific step in the viral life cycle are targeted. Over the past decade, significant research has been conducted toward the development of drugs against DENV. A few drugs that have shown *in vitro* inhibition of DENV replication include mefenamic acid, tetracyclines, amodiaquine, and chloroquine (Wong et al., 2016). Due to the similarity between ZIKV and DENV, much of the knowledge-base for DENV drug discovery can potentially be applied for the development of anti-ZIKV therapeutics (Weaver et al., 2016). To date, no ZIKV drug screening studies have been published. Nevertheless, the most challenging obstacle to overcome in the field of drug development would be the search of therapeutics for infected pregnant women (Lazear and Diamond, 2016).

### Preventative strategies

Collective responsibility and engagement for integrated vector management, particularly through the removal of stagnant water and use of insecticides (diethyltoluamide/ethyl butylacetylaminopropionate)/larvicides, is greatly emphasized due to the lack of vaccines and antiviral therapeutics against ZIKV (Lazear and Diamond, 2016; Pan American Health Organization, 2016a). Although, *A. aegypti* and *A. albopictus* are primarily responsible for the current ZIKV outbreak, vector control strategies and vector-pathogen interaction of all possible mosquito species are advised to be considered owing to ZIKV's ability to evolve (Ayres, 2016). It is also recommended to wear long-sleeved shirts and long pants, even potentially insecticide-impregnated clothing, in order to minimize vector contact (Basarab et al., 2016; Weaver et al., 2016). In addition, men who have a recent travel history to ZIKV endemic regions are advised to refrain from having unprotected sexual intercourse with their pregnant partner (Oster et al., 2016). For asymptomatic travelers returning from ZIKV endemic regions, barrier contraception for 28 days is recommended (Wong et al., 2016).

The unexpected potential link between ZIKV infection and microcephaly has resulted in increased prenatal surveillance in ZIKV endemic regions (Lazear and Diamond, 2016). Currently, it is recommended that public health authorities in ZIKV endemic regions provide access to contraceptives, prenatal care, and safe abortion services. Efforts toward educating the population, particularly in ZIKV endemic regions and travelers, regarding the potential routes of ZIKV transmission and preventative measures should be greatly emphasized (Lazear and Diamond, 2016). Increased vigilance toward imported cases of ZIKV infection and increased surveillance of individuals returning from ZIKV endemic regions would most certainly reduce autochthonous ZIKV transmission and global spread (Marano et al., 2016). In addition to the current existing surveillance systems, more emphasis should also be put into appropriate ZIKV diagnosis and monitoring of the potentially associated teratogenic and neurological complications (Pan American Health Organization, 2016a). Standard healthcare precautions should also be taken to eliminate mosquitoes from healthcare facilities in order to prevent autochthonous ZIKV transmission (Wong et al., 2016).

#### Entomological surveillance

Entomological surveillance allows for early detection of a potential virus outbreak, vector distribution and density, and evaluation of vector control strategies (Basarab et al., 2016). Faye et al. (2014) have designed a specific, rapid, and sensitive one-step qRT-PCR assay (primers targeting the ZIKV NS5 gene) for the fast detection of mosquito-originated ZIKV isolates from Africa and Asia. Several new technologies have shown to be promising for vector control in ZIKV endemic regions or upon detecting ZIKV positive mosquitoes in a new region (Yakob and Walker, 2016). The most simplified and time and cost-efficient strategy for reducing the mosquito population is through the introduction of lethal mosquito traps. According to a study by Barrera et al. (2014), implementation of lethal traps in two urban areas in Puerto Rice resulted in approximately 50–70% reduction of *A. aegypti* mosquitoes. A more technical approach would be through the introduction of genetically modified male mosquitoes carrying a dominant lethal gene expressed at the larval stage which causes death in offspring upon mating with wild female mosquitoes (Wise de Valdez et al., 2011). Although, this approach has the potential to significantly reduce the mosquito population, scaling up may be technically and financially challenging (Weaver et al., 2016). Another potential strategy, which has shown positive potential for DENV control, is through the use of the endosymbiotic relationship between *Aedes* mosquitoes and the *Wolbachia* bacteria. The endosymbiotic relationship interferes with virus replication in the mosquitoes (inhibitory effect). However, the potential of the virus to evolve and overcome the inhibitory effect of the endosymbiotic relationship must be taken in to consideration (Ritchie et al., 2015).

## Conclusions

The recent ZIKV outbreaks in the Americas have raised alarming concerns regarding the possible association of ZIKV infection with unexpected clinical manifestations, such as prenatal microcephaly and GBS. The risks and severity of ZIKV infection have been difficult to evaluate due to the poor understanding of ZIKV transmission dynamics and the absence of standardized ZIKV detection technique. To this end, evidence from published reports suggesting the potential association of ZIKV infection with prenatal microcephaly and GBS have been summarized in this review. In addition, this review discussed the current advances in ZIKV transmission and detection and emphasized the importance of understanding transmission dynamics for the subsequent development of a rapid cost-effective and time-efficient ZIKV detection assay and control strategy. Lastly, strong emphasis on the implementation of stringent surveillance systems (for humans and mosquitoes) as a preventive strategy is advised, particularly in tropical regions where the potential for ZIKV outbreak is more likely.

Further studies could investigate the association between ZIKV infection and microcephaly/GBS through case-controls studies in order to rule out potential etiological confounding factors. Future studies could also explore other potential ZIKV reservoirs and further investigate the ZIKV pathogenesis pathways and host cellular response to aid the development of a robust detection assay, ZIKV vaccine, and antiviral therapeutics.

## Author contributions

AS collected data, compiled data and wrote the manuscript, SL edited and reviewed the manuscript.

### Conflict of interest statement

The authors declare that the research was conducted in the absence of any commercial or financial relationships that could be construed as a potential conflict of interest. The reviewer CMGDF and handling Editor declared their shared affiliation and the handling Editor states that the process nevertheless met the standards of a fair and objective review.

## Abbreviations

CHIKV: Chikungunya virus
DC-SIGN: Dendritic Cell-Specific Intercellular adhesion molecule-3-Grabbing Non-integrin
DENV: Dengue virus
ELISA: Enzyme-Linked Immunosorbent Assay
ER: Endoplasmic reticulum
GBS: Guillain-Barré Syndrome
HPV: Human papillomavirus
HSV: Herpes simplex virus
Ig: Immunoglobulin
JEV: Japanese encephalitis virus
NASBA: Nucleic acid sequence-based amplification
NS5: Non-structural viral protein 5
PM: Prenatal microcephaly
PRNT: Plaque reduction neutralization test
qRT-PCR: Real time quantitative RT-PCR
RT-PCR: Reverse transcription polymerase chain reaction
RT-PCR: Reverse transcription polymerase chain reaction
TBEV: Tick-borne encephalitis virus
UTR: Untranslated region
WHO: World Health Organization
WNV: West Nile virus
YFV: Yellow Fever virus
ZIKV: Zika virus.
